# Kidney Diseases Caused by Complement Dysregulation: Acquired, Inherited, and Still More to Come

**DOI:** 10.1155/2012/695131

**Published:** 2012-11-14

**Authors:** Saskia F. Heeringa, Clemens D. Cohen

**Affiliations:** ^1^Division of Internal Medicine, University Hospital Zurich, Raemistrasse 100, 8006 Zurich, Switzerland; ^2^Division of Nephrology, University Hospital Zurich, Raemistrasse 100, 8006 Zurich, Switzerland

## Abstract

Inherited and acquired dysregulation of the complement alternative pathway plays an important role in multiple renal diseases. In recent years, the identification of disease-causing mutations and genetic variants in complement regulatory proteins has contributed significantly to our knowledge of the pathogenesis of complement associated glomerulopathies. In these diseases defective complement control leading to the deposition of activated complement products plays a key role. Consequently, complement-related glomerulopathies characterized by glomerular complement component 3 (C3) deposition in the absence of local immunoglobulin deposits are now collectively described by the term “C3 glomerulopathies.” Therapeutic strategies for reestablishing complement regulation by either complement blockade with the anti-C5 monoclonal antibody eculizumab or plasma substitution have been successful in several cases of C3 glomerulopathies. However, further elucidation of the underlying defects in the alternative complement pathway is awaited to develop pathogenesis-specific therapies.

## 1. Introduction

The central function of the kidney for whole body homeostasis is based on adequate blood flow and pressure, sufficient glomerular capillary surface for selective filtration, and subsequent secretion and reabsorption of solutes in the tubular system. The essential role of the glomerulus as a filtration unit can be estimated by the fact that most diseases leading to chronic kidney disease and end-stage renal disease with the need for dialysis or transplantation are caused by glomerulopathies. The glomerulus as a specialized capillary convolute is prone to any vascular damage and is affected as part of a generalized microangiopathy in common diseases such as diabetes mellitus or arterial hypertension. However, the glomerulus can also be affected by specific circulating factors, including antibodies against glomerular antigens, circulating immune complexes, or activated factors of a dysregulated complement system. 

The complement system as an essential component of the innate immune system plays an indispensable role in the elimination of invading microorganisms as a first line of defense [1, 2]. Furthermore, the complement system bridges innate and adaptive immunity. The cross-talk between toll-like receptors—as another key component of the innate immune system—and the complement system has been a key aspect of research as of their synergistic interaction to increase activation of inflammatory responses [3]. Complement activation runs through three major pathways (classic, alternative, and mannose-binding lectin) that all generate the enzyme complex C3-convertase which cleaves C3 into C3a and C3b. Herein, four main activation steps are distinguished: initiation of activation, activation and amplification of C3-convertase, activation of C5-convertase, and activation of the terminal pathway activity which is characterized by the assembly of the membrane attack complex (MAC) [4]. Importantly, the alternative pathway is constantly activated at low levels. Cascade progression and activation, however, is strictly controlled by complement regulating proteins such as complement factor H (CFH) and complement factor I (CFI): the two most important inhibitory proteins of the alternative pathway. 

**Figure 1:**
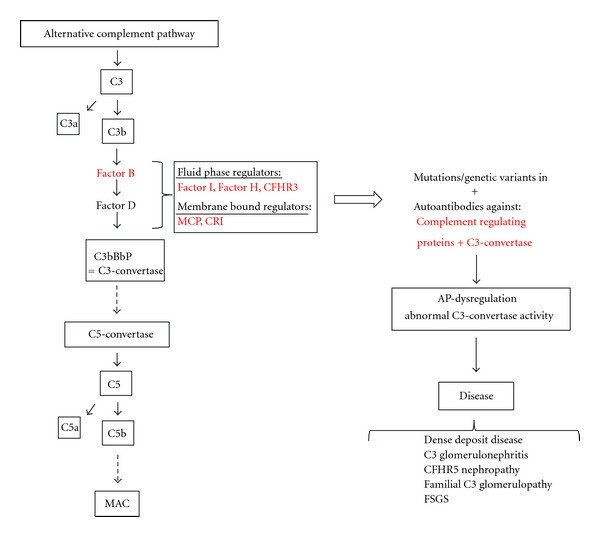
Dysregulation of the alternative complement cascade due to acquired or genetic factors leads to defective complement control causing a range of complement-associated glomerulopathies. C3 is cleaved to generate C3a and C3b. After binding of C3b to factor B, the complex is cleaved by factor D to form C3-convertase. The initial convertase constantly cleaves C3 at a low rate (referred to as “tick-over” of the alternative pathway). Binding of another C3b-fragment to C3-convertase creates a C5-convertase after which the pathway proceeds in the same manner as the classical pathway recruiting additional complement factors to ultimately form the membrane attack complex (MAC). The alternative pathway is strictly regulated by complement regulating proteins (listed in red). Mutations, genetic variations, or antibodies against complement regulating proteins or C3-convertase lead to abnormal C3-convertase activity. The subsequent deposition of activated complement products causes a range of complement-associated glomerulopathies. Abbreviations: C3; complement component 3, CFHR3; complement factor H related protein, AP; alternative pathway.

Complement dysregulation has been early recognized to be a central event in many nephropathies, and peripheral markers for complement activation (especially serum levels of C3 and C4) are tested routinely for different acquired renal diseases, for example, postinfectious glomerulopathy and proliferative lupus nephritis, glomerular capillaritis due to cryoglobulinemia or cholesterol embolism. Moreover, an increasing number of inherited renal diseases and renal diseases due to acquired factors with genetic predisposition for complement dysregulation are discovered such as atypical hemolytic uremic syndrome (aHUS) and membranoproliferative glomerulonephritis (MPGN) forms including dense deposit (DDD), C3 glomerulonephritis (C3GN) and CFHR5 nephropathy (Figure 1) [5, 6]. Mutations in *CFH* leading to CFH dysfunction and subsequently aHUS are the best known disease-causing mutations, but mutations in several other genes coding for complement factors and regulatory proteins have been identified in complement-related glomerulopathies (e.g., *C3, CFI, CFHR1-5, MCP *(membrane cofactor protein)). Genome-wide linkage analysis recently added novel polymorphisms and disease-causing mutations in complement genes to the list of hereditary complement-related nephropathies [7, 8]. In the following minireview we give an overview of complement-related glomerulopathies. Atypical HUS, a syndrome with prominent nonrenal, that is hematological and neurological manifestations, will not be discussed.

## 2. C3 Glomerulopathy

Patients with MPGN due to complement dysregulation manifest with proteinuria, (micro)hematuria and a variable degree of renal insufficiency. Kidney biopsy results show an altered glomerular basement membrane with double contours mostly due to subendothelial deposits, besides a variety of additional alterations such as hypercellularity and additional deposits [6]. As MPGN can be immune-complex-mediated, specific immunofluorescence has to be employed when evaluating the renal biopsy to differentiate between immunglobulin-mediated MPGN and complement-mediated MPGN.

Based on the localization of deposits in electron microscopy, MPGN has been classified into three different types: type I (subendothelial deposits), type II (intramembranous deposits), and type III (subendothelial and subepithelial deposits) [9]. Type I and III typically are immunglobulin-mediated diseases caused by the deposition of immune complexes as a result of for example circulating immune complexes, monoclonal gammopathies or chronic infections. MPGN II which is also called dense deposit disease, is characterized by complement component 3 (C3) containing dense deposits in the glomerular basement membrane that are a result of a dysregulation of the complement alternative pathway. As the inflammation is caused directly by the deposition of complement products, immunoglobulins are not involved and therefore not observed in immunofluorescence studies [6]. 

The subgrouping of MPGN has led to some confusion as all types of MPGN stain positive for C3 but immunglobulin staining can be negative even in some cases of MPGN I and III. Sethi et al. therefore proposed a classification driven by the findings on immunofluorescence, classifying MPGN as either immunoglobulin positive or negative [10]. Hence, immunoglobulin-negative but C3-positive MPGN is newly referred to as C3 glomerulopathy. Examples of C3 glomerulopathies are C3GN and DDD that can be distinguished by electron microscopical findings (see Table 1).

C3GN appears to be a key example of a dysregulated alternative and terminal complement pathway in which the deposition of complement is triggered despite the absence of antibody deposition [5, 10, 11]. Besides the identification of several disease-causing mutations in alternative pathway inhibitors, some autoantibodies leading to the activation or blockage of alternative pathway proteins have also been identified as a cause of C3GN. In a recent study by Servais et al., patients with C3GN (and additional patients with other forms of MPGN) were screened for mutations and rare variants in *CFH*, *CFI,* and *MCP* [12]. Although genetic abnormalities found in patients with C3GN were similar to the ones reported in individuals affected by aHUS, the genetic background predisposes specifically for the respective clinical and histological phenotype [12]. A rare, recently described variant of C3GN is CFHR5 nephropathy, a monogenic disease caused by mutations in the gene encoding complement factor-related protein 5 (CFHR5) [7]. CFHR5 is structurally related to CFH and possibly acts as a cofactor inhibiting C3-convertase [13]. In a cohort of patients with familial CFHR5 nephropathy sharing the same founder mutation, it was shown that the phenotype-spectrum among family members is broad [14]. As of this phenotypic heterogeneity, it is assumed that other factors like predisposing modifier genes and environmental factors as complement-activating infections also play a role in the development and phenotype of disease. CFHR5 is a member of the CFH related protein family, consisting of 5 proteins; CFHR1-5. Little is known about the function of these proteins, but there is increasing evidence that these protein families may either be involved in disease development or protection from complement dysregulation, respectively. CFHR1 inhibits C5-convertase activity and the formation of the terminal complex. CFHR3 also has complement regulatory activity as it inhibits C3-convertase [15]. Interestingly, a complete absence of both genes (Δ*CFHR3-1)* is not uncommon in the normal population. The deletion of *CFHR3-1* has even been associated with protection from both complement- and age-related macular degeneration as well as IgA nephropathy, the most common mesangioproliferative glomerulonephritis with prominent mesangial IgA and secondary local complement activation [16]. In a recent study, a hybrid *CFHR3-1* gene was shown to cause familial C3 glomerulopathy [17]. The authors suggested a possible dominant mechanism of this genetic alteration leading to an increased expression of both proteins, interfering with complement processing and leading to accumulation of C3 [17]. 

Dense deposit disease (DDD) is closely related to C3GN and recent data suggest that both may represent extremes in a continuous spectrum of complement-related MPGNs [6, 12]. Both diseases show similar features in light and immunofluorescence microscopy and they are distinguished by electron microscopy. Here, C3GN is characterized by mesangial, subendothelial, and intramembranous deposits, whereas DDD is characterized by osmophilic dense deposits along the glomerular and tubular basement membranes [18, 19]. By an advanced mass spectrometry approach on glomerular isolates, Sethi et al. detected activated components of the alternative pathway and of the terminal complement pathway in patients with DDD [20]. Because of a very high recurrence rate after kidney transplantation, a systemic cause of DDD has been early suggested. Hence, the identification of the first C3 nephritic factor (C3Nef) as an autoantibody that stabilizes C3-convertase, was a major achievement [21]. The presence of C3Nefs is the most common association with alternative pathway dysregulation in DDD [22]. Binding of C3Nef to the alternative C3-convertase increases its half-life leading to uncontrolled alternative pathway activation and a massive consumption of C3. However, C3Nef activity is not always associated with low C3 levels in plasma and C3Nef is not specific for DDD as it can be found in other diseases as well as in healthy individuals [23]. Less common causes for DDD are inhibitory CFH autoantibodies, CFH deficiency, or functional CFH-defects, the latter both due to genetic mutations leading to reduced CFH activity. Mutations that lead to a complete CFH deficiency are rare though, and most functional CFH defects go along with normal CFH levels in plasma [24, 25]. Interestingly, not all patients with a functional CFH defect develop DDD, as CFH deficiency can also lead to aHUS and not all individuals with similar genetic variants develop the same phenotype [8]. 

In order to analyze the causes of alternative pathway dysregulation, several groups studied DDD cohorts employing functional and genetic tests [12, 22]. In these studies, a probable cause for complement dysregulation could be identified in about 80% of DDD patients [12, 22]. Detection of C3Nef was the most frequent single finding, but in some patients also autoantibodies against CFH or CFB were detected [22]. Gene variants in *CFH* were detected in 10–17% of DDD patients [12, 22]. Notably, a functional CFH defect frequently coexisted with the presence of C3Nef. Similarly, in C3GN the detection of C3Nef was the most common complement abnormality found in 45% patients with C3GN that were screened by Servais et al. [12]. Besides C3Nef, additional autoantibodies against CFH, CFB and to the individual components of C3-convertase (C3b and factor B) have been described in C3 glomerulopathies [12, 18, 26–28]. Anti-factor B autoantibody for example was found in a patient with DDD that was able to bind and thereby stabilize C3-convertase leading to an increased consumption of C3 [28]. This again indicates that DDD and C3GN have many pathogenetic and histological aspects in common and may represent extremes of a continuum.

## 3. Treatment

In order to decrease proteinuria and improve blood pressure control, nonspecific treatment with angiotensin converting enzyme (ACE) inhibitors or angiotensin type II receptor blockers is recommended in all patients. With improved understanding of the pathogenesis of C3 glomerulopathies more specific therapies could be applied. In patients with C3Nef or autoantibodies to CFH or CFB immunosuppressive therapies including rituximab or plasma exchange have been reported to slow disease progression or to help to avoid recurrence after transplantation [19, 26]. 

With the increasing knowledge about the underlying mechanisms and the specific complement defects in C3 glomerulopathies, specific complement-targeting therapies have been successfully applied in several cases of DDD and C3GN. As can be expected, large-scale clinical studies are missing in these rare diseases. Especially eculizumab, a humanized anti-C5 monoclonal antibody, represents a promising agent as it blocks C5b-9 formation, the terminal event in the complement cascade. The antibody has been approved by both the U.S. Food and Drug Administration as well as the European Commission for the treatment of paroxysmal nocturnal hemoglobinuria and, more recently, atypical HUS. Eculizumab was even suggested to be an effective agent in children with enterohemorrhagic *Escherichia coli* (EHEC) infection-caused HUS [29]. However, this was not evident in a case-control study reporting a mainly middle-aged population of a recent outbreak of EHEC induced HUS in northern Germany [30]. But for DDD and MPGN with CFHR1 deficiency, recent anecdotic reports suggest a treatment effect of eculizumab with stabilization of kidney function, decrease in proteinuria [31, 32], and even improvement in histopathological findings [33]. In a recent study reporting of three subjects with DDD and three subjects with C3GN who were treated with eculizumab for one year, response to treatment was seen in some but not all patients. Elevated serum membrane attack complex normalized on therapy, serum creatinine improved and proteinuria was reduced [34]. A very recent pathology report on C3GN patients treated with eculizumab showed de-novo monoclonal staining for IgG-kappa in the same distribution as C3 and C5b-9 in all posttreatment protocol biopsies, indicating binding to C5 and glomerular deposition of eculizumab [35]. As the authors state, the long-term clinical significance of these therapy-induced immune deposits together with apparent drug-tissue interactions is not known. Beside eculizumab as target-specific but costly treatment option fresh frozen plasma (FFP) infusions were given to several cases with functional CFH deficits and resulted in the prevention of further disease progression [36, 37]. In a recent case report of two unrelated patients with MPGN and MPGN II with combined autoantibodies for factor B and C3, one patient received immunosuppressive treatment leading to a significant decrease of both autoantibodies [26].

In order to guide such disease specific treatment, it may become important to evaluate the alternative complement pathway more comprehensively in patients that have a renal biopsy consistent with C3 glomerulopathy. Complement proteins (CFH, CFI, CFB), complement degradation products (C3c, C3d), soluble serum membrane attack complex (sMAC), and disease associated autoantibodies (C3 nephritic factor, anti-factor H, anti-factor B, anti-C3b [26]) might expand the diagnostic arsenal of complement specific markers in the future. 

## 4. Conclusions

In sum, the spectrum of renal disease phenotypes due to complement dysregulation is diverse. And it is still increasing: in a recent case report, Sethi et al. reported of single nucleotide polymorphisms in genes encoding CFH and C3 to be linked to the development of focal segmental glomerulosclerosis (FSGS), potentially extending the involvement of complement dysregulation to “podocytopathies” [38, 39]. Interestingly, this finding is in line with data from the European Renal cDNA Bank indicating alteration of intraglomerular transcript levels of complement-related gene products in FSGS (*own unpublished observation*). 

As outlined above, several independent approaches such as Mendelian genetics, genome-wide association studies, transcriptomic and proteomic approaches as well as histopathology and functional studies underline the relevance of complement dysregulation in several kidney diseases, which currently undergo a redefinition. With the increasing insight into the pathophysiology, more specific complement targeting therapies may become available for the treatment of both ultrarare and more frequent complement-associated renal diseases. 

## Figures and Tables

**Table 1:** C3 glomerulopathies.

Diseases	EM-findings	Alternative pathway abnormalities	Disease specific treatment options
Dense deposit disease	(i) Osmophilic wavy dense deposits within GBM, mesangial matrix, tubular BM	(i) Autoantibodies (C3Nef, FHAA, FBAA, C3-convertase AA) (ii) Mutations/genetic variations (*CFH, CFI, CFB, MCP*)	(i) Infusion of fresh frozen plasma (ii) Plasmapheresis (iii) Eculizumab (iv) Immunosuppressive treatment (in case of autoantibodies)
C3 glomerulonephritis	(i) Mesangial, subendothelial, subepithelial and intramembranous deposits	(i) Mutations/genetic variations (*CFH, CFI, MCP*) (ii) Autoantibodies (C3Nef, FHAA)	(i) Eculizumab (ii) Immunosuppressive treatment (in case of autoantibodies)
CFHR5 nephropathy	(i) Mesangial, subendothelial, subepithelial deposits	(i) *CFHR5*-mutation	(i) No treatment of proven efficacy (ii) Plasma exchange associated with good outcome
Familial C3 glomerulopathy	(i) MPGN type III (ii) Subendothelial, subepithelial deposits	(i) Familial hybrid *CFHR3-1* gene autosomal dominant inheritance	(i) No treatment of proven efficacy

Abbreviations: C3: complement component 3, CFHR5: complement factor H related protein 5, CFH: complement factor H, CFI: complement factor I, MCP: membrane cofactor protein, FHAA: factor H autoantibody, FBAA: factor B autoantibody, (G) BM; (glomerular) basement membrane, MCP: membrane cofactor protein.
